# Anxiety in women referred for colposcopy: a prospective observational study

**DOI:** 10.1007/s00404-021-06337-8

**Published:** 2021-11-26

**Authors:** Julia Wittenborn, Lisa Wagels, Tomas Kupec, Severine Iborra, Laila Najjari, Elmar Stickeler

**Affiliations:** 1 Department of Gynecology and Obstetrics, University Hospital of the RWTH Aachen, Pauwelsstrasse 30, 52074 Aachen, Germany; 2 Department of Psychiatry, Psychotherapy and Psychosomatics, University Hospital of the RWTH Aachen, Pauwelsstrasse 30, 52074 Aachen, Germany; 3 Research Center Jülich, JARA Institute Brain Structure Function Relationship Institute for Neuroscience and Medicine (INM-10), 52425 Jülich, Germany

**Keywords:** Anxiety, STAI, Cervical cancer screening, Cervical intraepithelial neoplasia

## Abstract

**Purpose:**

To evaluate the occurrence of anxiety in women attending a colposcopic examination within the new cervical cancer screening in Germany.

**Methods:**

One hundred and fifty-six patients were asked to fill out Spielbergers STAI inventory form prior to their colposcopic examination. For the statistical analysis, a two by two between-group design was applied including the following group factors: the repeat factors included patients, who presented to our centre of dysplasia for the first time (new) and patients who have had an examination in our centre before (repeat). Further, the factor diagnosis included two groups: first, patients with cervical dysplasia and second, patients with vulva diseases.

**Results:**

The analysis of the STAI results showed that patients presenting with cervical dysplasia for the first time had the highest levels of anxiety, directly followed by new patients in the vulva group. The ANOVA revealed a main effect of the repeat factor, *F*(1,140) = 7.53, *p* = 0.007. There was no significant effect of diagnosis.

**Conclusion:**

Regardless of the diagnosis, patients being transferred for a colposcopy within the cervical cancer screening program for the first time have very high anxiety levels. The prospect of a potentially painful examination seems to be a key factor. Only a scientific evaluation of the new cervical cancer screening will be able to show if the rising numbers of colposcopic examinations is really worth the risk of exposing so many more women to the emotional distress of a colposcopy.

**Supplementary Information:**

The online version contains supplementary material available at 10.1007/s00404-021-06337-8.

## Introduction/background

Colposcopy is the most important examination in the work-up of suspicious cervical cytologies. It is a visualization of the cervix using a stereoscopic binocular microscope with low magnification. Thanks to the introduction of the cervical cancer screening program in the 1970s, today cervical cancer is a rare disease in Germany [1]. Only about 4300 patients are diagnosed with cervical cancer every year [2]. The incidence rates of cervical intraepithelial neoplasias (CIN 1–3) are 50–100 times higher and often require colposcopic evaluation with the aim to detect cervical cancer precursors [3]. With the introduction of the new cervical cancer screening program in Germany in January 2020 colposcopies have become even more frequent and pose a mandatory examination even for patients with one-time cervical abnormalities in case they carry a HPV high-risk infection [4]. Little attention has been paid to the psychological effects of these investigations and their results, and gynaecologists are often unaware that there may be many untoward effects [5].

Anxiety is characterized by excessive worrying and physical symptoms originating due to heightened activation of the sympathetic nervous system [6]). Anxiety can greatly impair a person’s cognitive abilities [7, 8] and cause a negative cognitive bias [9]. Thus, it is posing a threat to the quality of life and well-being. Anxiety also decreases the ability to recall and act on advice, making women less likely to comply with the information they are given [10].

A high level of anxiety before and during colposcopy is a clinical phenomenon that has been described before [11–14]. Even different interventions to reduce patients anxiety have been investigated, but with no clear benefit for the patients. Neither information leaflets, booklets, video presentations nor the playing of soothing music was able to reduce patients anxiety [13, 15–17]. Thus leaving a big question mark to the possible reasons and the best way for reducing the observed high levels of anxiety.

Here, we present the results of our prospective observational study of anxiety in women referred to our centre of dysplasia at the university hospital in Aachen. Anxiety levels of all referred patients were assessed, thus enabling a comparison between patients presenting for the first time (new) and those who have experienced a colposcopic examination before (repeat). Additionally, we aimed to investigate differences in anxiety by age group, by different reasons for referral to colposcopy or by the severity of the cytological abnormalities.

## Materials/methods

### Study design

This prospective, observational study was carried out at the university hospital in Aachen, Germany during a period of 3 months in patients referred to the centre of dysplasia for colposcopic evaluation to assess the level of anxiety in these patients.

### Study participants

All patients referred for colposcopic evaluation in our centre of dysplasia from August 2020 till October 2020 were asked to fill out Spielbergers STAI inventory form along with the standard form for taking patients medical history prior to the examination. All patients who filled out the form with no more than 2 blanks were included in the study. Exclusion criteria were age < 18, STAI form incomplete (more than two blanks), insufficient knowledge of the German language to fill out the form, patients with diseases other than cervical dysplasia or vulvar diseases.

### Data collection

The STAI questionnaire is a self-evaluation questionnaire with 20 questions. All items are rated on a 4-point Likert-type scale (e.g., from “Almost Never” to “Almost Always”), thus the scores range from 20 to 80. The scale has 10 reverse-scored items. The essential qualities evaluated by the STAI-State Anxiety scale are feelings of apprehension, tension, nervousness, and worry. Higher scores indicate greater anxiety.

The Spielberger State-Trait Anxiety Inventory (STAI) is the most frequently used measure of state and trait non-disorder-specific anxiety with a citation index over 16,000 since its first publication [18]. The questionnaire has been translated into 48 languages as of 2011. The STAI has been shown to have excellent psychometric properties with good reliability and validity [19]. Various tests have been conducted on the STAI and have provided sufficient evidence that the STAI is an appropriate and adequate tool for studying anxiety in research and clinical settings [20].

### Ethical considerations

Participation in the study was voluntary and all procedures were in line with the declarations or Helsinki (2013) and its later amendments. The study was approved by the local ethics committee (EK 350/20).

### Statistical analysis

For the statistical analysis, a two by two between-group design was applied including the following group factors: the repeat factors included patients, who presented to our centre of dysplasia for the first time (new) and patients who have had an examination in our centre before (repeat). Further, the factor diagnosis included two groups: first, patients with cervical dysplasia and second, patients with vulva diseases. Patient groups were compared regarding age in a two by two between-subject ANOVA. The main analysis was conducted as an ANCOVA, with the same two by two design including age as a covariate. As a secondary analysis within the cervix group only, the severity scores were tested as potential influence factor for anxiety by conducting a correlation analysis (Spearman’s Rho).

All data were analysed using IBM SPSS statistics 26. TP-values < 0.05 were considered significant and Bonferroni correction was applied for post-hoc comparisons.

## Results

**Fig. 1:**
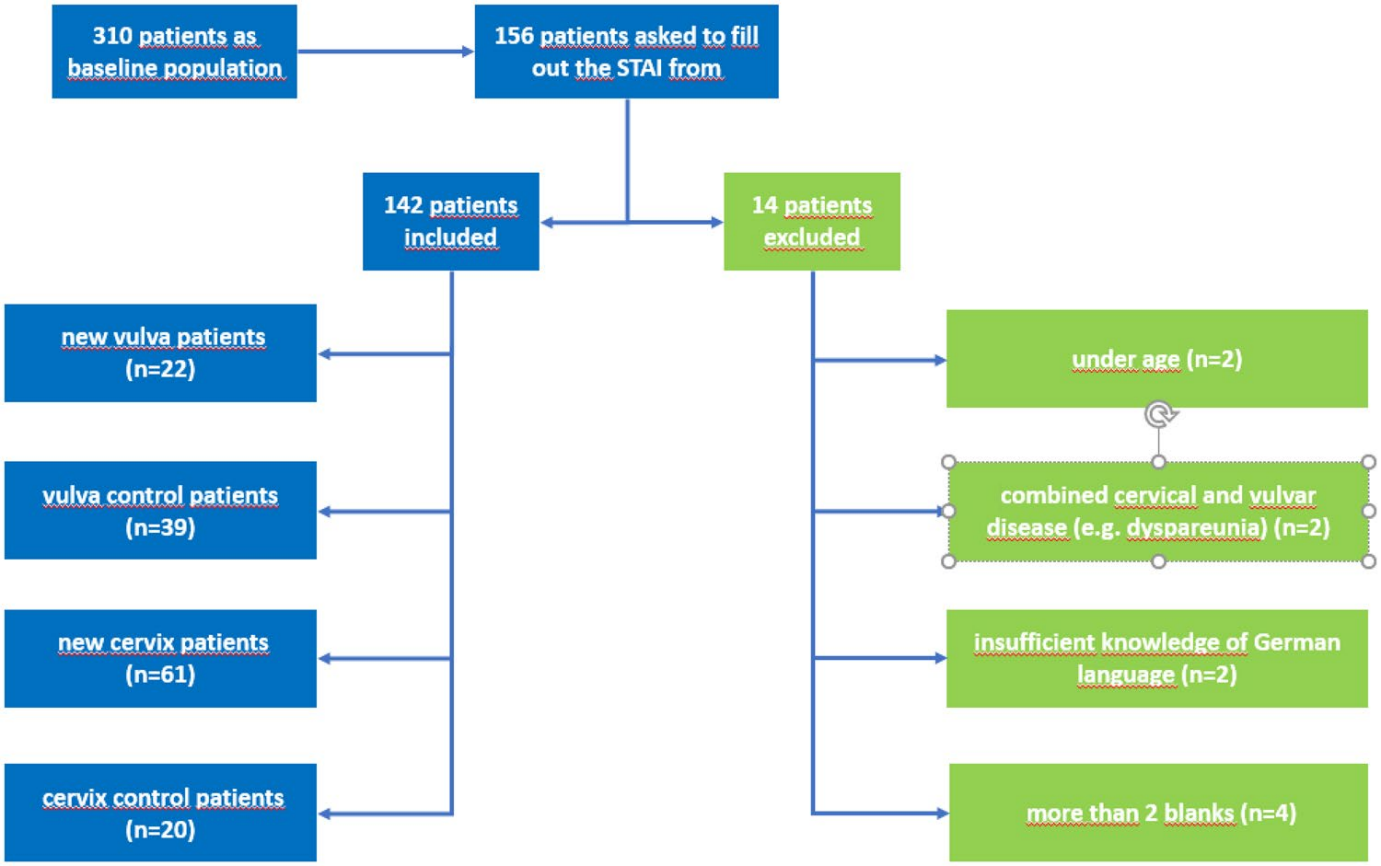
Flow chart

Of the 156 patients who were asked to fill out the form, 142 were included in the study (Fig. 1). The patients characteristics are listed in Table 1. Mean age differed between the cervical dysplasia group and the vulva group, *F*(1,141) = 6.61, *p* = 0.011, but did not differ with regard to the factor repeat, *F*(1,141) = 1.40, *p* = 0.240. There was no interaction between both group factors with regard to age, *F*(1,141) = 1.14, *p* = 0.288. The majority of patients in both groups of cervix patients (New and Repeat) were referred because of a cytological result of category III (HSIL, LSIL, ASC-H, AGC). In this group, patients were diagnosed with intraepithelial lesions or normal tissue biopsies. No cervical cancer was found. This also applies to patients with cytological results of category IV.

**Table 1:** patient characteristics

	New patients with cervical dysplasia (Group 1)	Control patients with cervical dysplasia (Group 2)
Age (mean ± SD)	45.6 ± 13.5	43.7 ± 15.2
Total number	61	20
Cervical cytology result as reason for referral		
IIp (ASC-US)	5 (8.2%)	5 (25%)
III D, IIIp, IIIx, IIIg (HSIL, LSIL, ASC-H, AGC)	40 (65.6%)	6 (30%)
IV (HISL)	16 (9.8%)	5 (25%)
V	1 (1.6%)	3 (15%)
Other	0	1 (0.5%)

	New patients with vulvar diseases	Control patients with vulvar diseases
Age (mean ± SD)	47.5 ± 18.9	53.7 ± 15.2
Total number	22	39

**Fig. 2:**
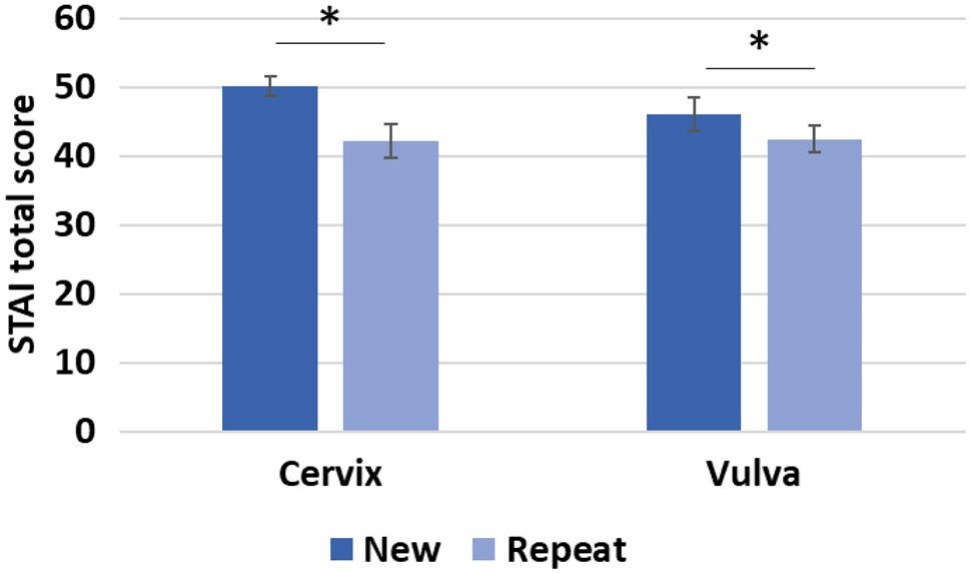
Mean and standard error of the STAI are depicted for each subgroup. Significant differences are indicated at *p* < 0.05

The analysis of the STAI results descriptively showed that patients presenting with cervical dysplasia for the first time (new patients) had the highest levels of anxiety, directly followed by new patients in the vulva group (Fig. 2). The ANOVA thus revealed a main effect of the repeat factor, *F*(1,140) = 7.53, *p* = 0.007. There was no significant effect of diagnosis, *F*(1,140) = 0.78 3, *p* = 0.379 and no significant interaction of diagnosis and repeat, *F*(1,140) = 1.03, *p* = 0.311. Age did not significantly influence anxiety levels, *F*(1,140) = 0.88, *p* = 0.349.

**Fig. 3:**
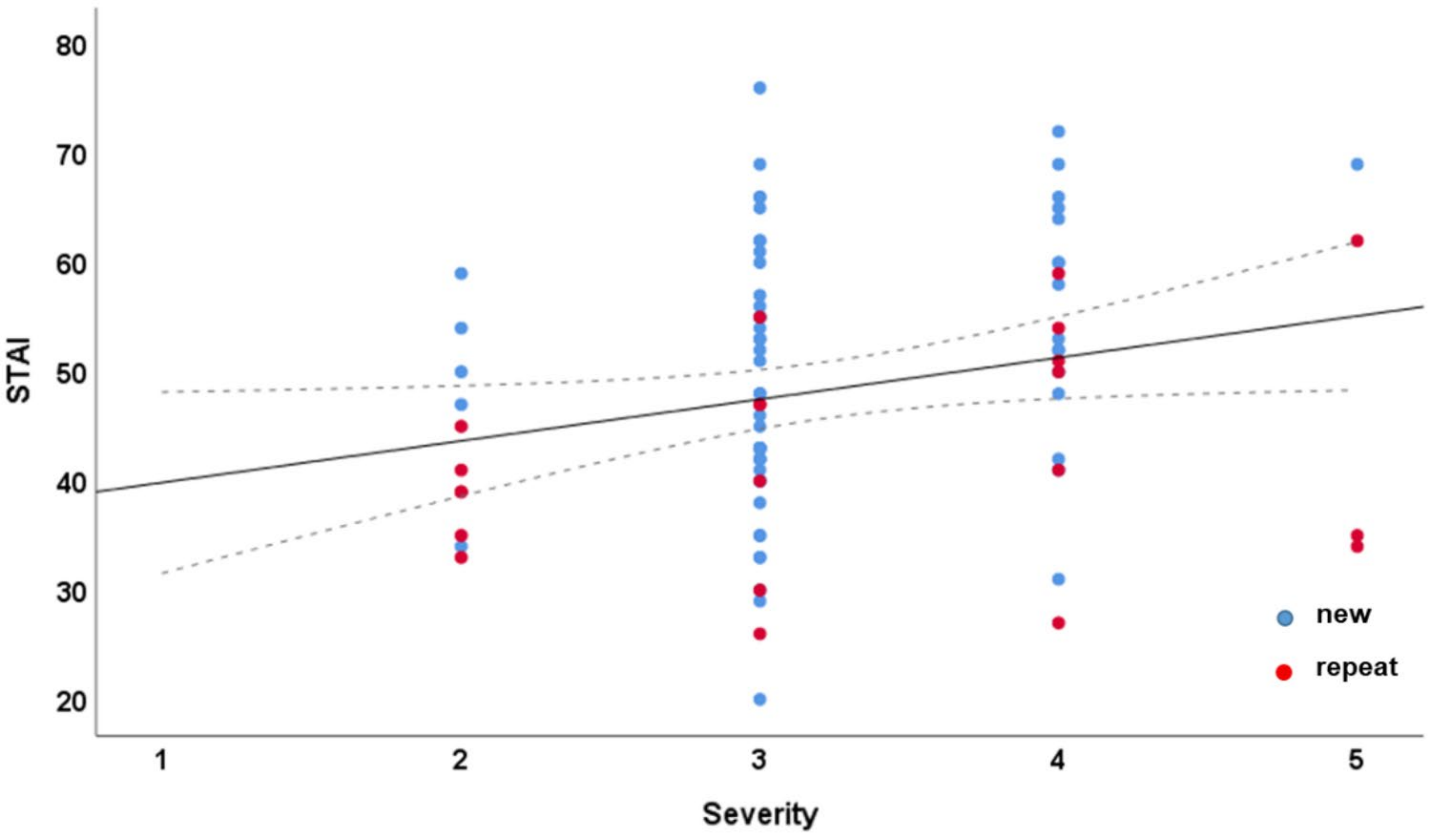
Scatter plot of the severity of cytological abnormalities upon referral and STAI for the cervix group. Regression line and confidence interval at 95% are indicated across the new (blue) and repeat (red) subgroup

The correlation analyses revealed that there was a significant correlation between the severity of cytological abnormalities upon referral to our centre of dysplasia and STAI in the cervix group (*n* = 84), ρ = 0.253, *p* = 0.020 (Fig. 3).

## Discussion

In this work, we were able to demonstrate that the levels of anxiety in women presenting for colposcopic evaluation are highest in patients presenting for the first time independent of the diagnosis. As colposcopic evaluations take a high priority in the new cervical cancer screening in Germany this work is highly relevant.

Looking into the literature one can find STAI anxiety levels in all kinds of situations. For example, STAI levels of patients undergoing open cardiac surgery within the next 24 h were at 40 (moderate anxiety) [21]. In women during the course of giving birth by caesarean section, highest STAI levels were measured at admission to the hospital and were 47.35 ± 10.83 [22]. No other health care associated situation was found in which STAI levels reached the observed “high anxiety” levels of the patient collective presenting for colposcopic evaluation for the first time.

We were able to show that the underlying diagnosis does not influence the occurrence of anxiety. It does not matter if the patients are stemming from the cervical cancer screening or are being referred for (mostly benign) vulvar diseases such as lichen sclerosus or condylomata accuminata. Thus, the often proposed fear of cancer as the main reason for high anxiety [12, 23, 24] levels cannot be backed by our study. The observed anxiety seems to be more strongly related to the anticipation of the procedure itself rather than the outcome.

This finding is in line with Mateau et al. who let women fill out a questionnaire before the colposcopy addressing the women’s main concerns. Most frequently, women stated that they feared a painful or uncomfortable examination [25].

There was no influence of age on the observed anxiety levels. This finding is in accordance with prior reports [12, 23]. We observed a main effect of the repeat factor, which means that regardless of the reason for referral for colposcopy patients had significantly lower anxiety levels in case they experienced a colposcopic examination before. Accordingly, Bosgraaf and colleagues’ focus group study found that psychological stress before colposcopy was caused by unsatisfactory explanations of abnormal smears and the colposcopy procedure itself [26].

Here, we see a great need for intervention, especially now, as the new cervical cancer screening in Germany with a main focus on colposcopy is getting started. It has been tried to reduce anxiety levels in women in the Netherlands by using video information presented to the patients five days prior to the examination, but it did not significantly reduce anxiety [17]. A Cochrane analysis reviewed measures to reduce anxiety on patients in England (e.g. information leaflets and video) and came to the conclusion that none was fit to reduce anxiety [15].

Our data suggest that an approach focused on detailed information about the examination itself may be more promising than giving the patients more information on the suspected diagnosis. Additionally, we suggest that the measured anxiety levels be taken into account upon the planned re-evaluation of the new cervical cancer screening in Germany [4]. Although there seems to be a slight correlation between the severity of the cytological result and the measured anxiety (Fig. 3), the observed levels were high throughout the entire patient population. Colposcopy is valuable in the investigation of women with abnormal PAP smear, but considerable disagreement as to whether women should be offered colposcopic screening in case of low-risk PAP smear or even normal PAP smear, in case they carry a HPV high-risk infection over a year, has arisen [27]. Our results should be considered upon the planned cost-effectiveness calculation of the new screening algorithm.

Our study has several strengths and limitations that need to be addressed. First, it is a prospective observational study with a good number of included patients and a clear, predefined goal to assess patients anxiety levels in the German cervical cancer screening. We implemented a control group of patients undergoing the same preliminary examinations and colposcopic examination, but who were not recruited within the cervical cancer screening.

Concerning the limitations, it is a monocentric study with a limited patient collective. Trait anxiety was not measured and can therefore not be excluded as a potential confounding variable. To draw final conclusions for the whole screening population, a multi-centre study would be necessary.

## Conclusion

Regardless of the diagnosis, patients being transferred for a colposcopic evaluation within the cervical cancer screening program in Germany have very high anxiety levels. The prospect of a potentially painful examination is probably a key problem. This issue should be aware to gynaecologists performing the cervical cancer screening. Only a scientific evaluation of the new cervical cancer screening will be able to show if the rising numbers of colposcopic examinations is really worth the risk of exposing so many more women even with low-grade cytological abnormalities or no abnormalities at all (HPV high-risk infection over one year) to the emotional distress of a colposcopic examination.

## Supplementary Information

Below is the link to the electronic supplementary material.

Supplementary file1 (PDF 228 KB)

## Data Availability

Not applicable.
