# Supplementary material for: Anxiety in women referred for colposcopy: a prospective observational study
Source: Arch Gynecol Obstet. 2021 Nov 26;305(3):625–30. doi: 10.1007/s00404-021-06337-8 (PMC8918141; doi:10.1007/s00404-021-06337-8)
Supplement: Supplementary file 1 — Supplementary file1 (PDF 228 KB) [file 404_2021_6337_MOESM1_ESM.pdf]

ETHIK-KOMMISSION AN DER MEDIZINISCHEN FAKULTÄT
DER RHEINISCH-WESTFÄLISCHEN TECHNISCHEN HOCHSCHULE AACHEN
Pauwelsstr. 30, 52074 Aachen - Tel. 0241-80 89 963 – FAX 0241-80 82 012
E-Mail: ekaachen@ukaachen.de

Ethik-Kommission an der Medizinischen Fakultät
der RWTH Aachen – Pauwelsstr. 30 – D - 52074 Aachen

Frau
Dr. med. Julia Wittenborn
Leitung Dysplasiezentrum der Frauenklinik
Im Hause
per E-Mail

Aachen, den 23.09.2020

Schmal/sp

Betrifft:

CTC-A-Nr.:	20-409
Protokoll-Nr:	-
Titel:	Anxiety in women with abnormal cervical cytology referred for colposcopy: a prospective observational study
Sponsor:	-
Eingereicht von:	Dr. med. Julia Wittenborn, Leitung Dysplasiezentrum der Frauenklinik, Uniklinik RWTH Aachen, Pauwelsstr. 30, 52074 Aachen
Antragsteller:	Dr. med. Julia Wittenborn, s. o.
LKP:	-
Lokaler Hauptprüfer:	Dr. med. Julia Wittenborn, s. o.
Internes Aktenzeichen:	EK 350/20

Hier: Stellungnahme

Sehr geehrte Fr. Dr. Wittenborn,

vielen Dank für Ihr Schreiben vom 10.09.2020 - Eingang in der Geschäftsstelle der Ethik-Kommission am 17.09.2020, in dem Sie uns um eine Stellungnahme zu dem von Ihnen geplanten Forschungsvorhaben bitten.

Es sollen Patientinnen, die aufgrund einer auffälligen Zervixzytologie oder eines auffälligen Kombinationstests (Zytologie+ HPV Test) im Rahmen der Zervixkarzinomvorsoge in Ihr Dysplasiezentrum überwiesen wurden, bei Erstanmeldung einen standardisierten Angstfragebogen (Spielbergers State-Trait-Angstinventar (STAI)) ausfüllen.

Sie versichern, dass die Datenerhebung mittels Fragebogen und Auswertung in anonymisierter Form erfolgt. Eine Antragstellung ist nicht erforderlich. Wir empfehlen allerdings eine Beratung durch den Datenschutzbeauftragten des UKA.

Es bestehen keine ethischen und berufsrechtlichen Bedenken gegen das Forschungsvorhaben.

Vorsitzender: Prof. Dr. G. Schmalzing; Geschäftsführerin: S. Pfaff (Fachärztin für Dermatologie): Tel. +49 241 80 85 330,
Fax +49 241 80 82 012; E-mail:ekaachen@ukaachen.de, Homepage: www.medizin.rwth-aachen.de/EK

Spezifischer Hinweis: Bitte beachten Sie, dass neben der hiermit vorliegenden zustimmenden Bewertung im Rahmen der berufsrechtlichen und berufsethischen Beratung durch die Ethik-Kommission vor Studienbeginn eine Datenschutzfolgeabschätzung (DSFA) oder Stellungnahme des hiesigen Informationssicherheitsbeauftragten vorliegen muss.

Die eingereichten Unterlagen und die derzeitigen Fragebögen wurden nicht im Rahmen einer Sitzung, sondern im Auftrag der Ethik-Kommission satzungsgemäß im vereinfachten Verfahren durch den Vorsitzenden und den stellv. Vorsitzenden der Ethik-Kommission bewertet.

Viel Erfolg bei Ihrem Forschungsvorhaben.

Mit freundlichen Grüßen

Prof. Dr. med. G. Schmalzing
Vorsitzender

PD Dr. med. R. Hausmann
Stellv. Vorsitzender

Die Ethik-Kommission ist nach Landesrecht konstituiert und bei den zuständigen Landesbehörden, beim Bundesamt für Arzneimittel (BfArM) sowie beim Bundesamt für Strahlenschutz (BfS) registriert. Sie berät unabhängig nach den Regeln des Weltärztebundes in der Deklaration von Helsinki über Forschung am Menschen in der Fassung von 1996 in Somerset West, nach nationalen Gesetzen, Vorschriften und der ICH-GCP-Leitlinie in der jeweils gültigen Fassung (siehe Homepage der Ethik-Kommission unter www.medizin.rwth-aachen.de/EK).

Vorsitzender: Prof. Dr. G. Schmalzing; Geschäftsführerin: S. Pfaff (Fachärztin für Dermatologie): Tel. +49 241 80 85 330,
Fax +49 241 80 82 012; E-mail:ekaachen@ukaachen.de, Homepage: www.medizin.rwth-aachen.de/EK
